# Local-Reference Patient Dose Evaluation in Conventional Radiography Examinations in Mazandaran, Iran

**Published:** 2016-06-01

**Authors:** D. Khoshdel-Navi, A. Shabestani-Monfared, M. R. Deevband, R. Abdi, M. Nabahati

**Affiliations:** 1 School of Medicine, Babol University of Medical Sciences, Babol, Iran; 2 Department of Medical Physics, Faculty of Medicine, Babol University of Medical Sciences, Babol, Iran; 3 Department of Medical Physics, School of Medicine, Shahid Beheshti University of Medical Sciences, Tehran, Iran; 4 Department of Radiology, Mazandaran University of Medical Sciences, Sari, Iran; 5 Department of Radiology, Babol University of Medical Sciences, Babol, Iran

**Keywords:** Conventional Radiography, Dosimeter, Patient Dose, Mazandaran, ESAK, ESD

## Abstract

**Background:**

The most efficient application of ionizing radiation is serving medical purposes and using this radiation has caused people to learn that artificial sources of radiation exposure among these resources can be of highest exposure rate.

**Obiective:**

The present study is aimed at initially establishing a baseline for local-reference dose level in Mazandaran, Iran in 12 projections of the most conventional x-ray examination.

**Methods:**

In this study, 13 public hospitals in Mazandaran province were selected for review and required data collected for ten adult patients with mean weight of 70±10kg in each projection. Then, information of each center was separately analyzed. Next, in order to measure x-ray output tube, the dosimeter RTI model Barracuda calibrated has been applied for measuring air karma within energy rage of 40-150kvp. ESAK and ESD parameters, usually used for monitoring DRL in conventional radiography, were calculated.

**Results:**

Mean ESDs in this study has been obtained to 1.47±0.98 for skull (PA/AP), 1.01±0.79 for skull (LAT), 0.67±0.38 for cervical spine (AP), 0.79±0.37 for cervical (LAT), 0.49±0.38 for chest (PA/AP), 1.06±0.44 for chest (LAT), 2.15±0.73 for thoracic spine (AP), 3±0.87 for thoracic spine (LAT), 2.81 ±0.82 for lumbar spine (AP), 4.28±0.78 for lumbar (LAT), 2.07±1.17 for abdomen and 1.90±0.99 for pelvis, respectively. The ESDs calculated for chest examination in both projections, PA and LAT are more than values recommended by the UK (2000), Brazil and Slovenia.

**Conclusion:**

The present study has determined wide variations in radiation dose of x-ray examinations among hospitals in Mazandaran, Iran. In order to reduce skin dose, an optimization procedure should be considered. Application of a reference dose (DRL) could be a practical method for this purpose. The role of optimization of radiography parameters for reducing patient dose is a significant issue. Through optimizing parameters, it would be possible to preserve image quality while reduction of patient dose.

## Introduction

There is no doubt that the most efficient application of ionizing radiation is for medical purposes and using this radiation has caused people to know that artificial sources of radiation exposure among these resources can be of highest exposure rate[1]. Compared to other imaging modalities such as MRI, CT scan and Digital Radiography, conventional radiography is still prevailing as an important and essential diagnostic tool in many developed countries. Through report of United Nations Scientific Committee on the Effects of Atomic Radiation, it has been announced that repetitive examinations over 5 years had approximately doubled even tripled in some countries. The report concluded that public exposure has been increased, especially in countries that still have not spread in maintenance of medical devices. Thus, due to public concern over increase in radiation dose levels for patients under diagnostic tests, X-rays were considered in contrast with the experiments with excellent benefit for the treatment of patients with dose[2, 3]. Usually, there are two concerns in diagnostic radiology as follows: the first concern is related to poor image quality that is not suitable for initial diagnostic information, which makes re-imaging of patients to increase unnecessary exposure frequency and waste of resources and money, especially in developed countries[4, 5]. The second concern is a significant difference in patient dose for the same test. National studies have been conducted in many countries across a wide variety of patient doses in similar diagnostic radiology[6, 7]. Hence, it is important to provide radiation protection and safety without underlay burdening physicians or limiting the benefits of medical radiation exposure.

In this regard, International Commission on Radiation Protection (ICRP) in 1982 stated that dose for a specific examination in patients with other similar hospitals varies equal to 2-10 times[8]. The amount of patients dose in a hospital has shown factor of 20 or more from room to room[6, 7, 9]. This has made various regulatory organizations to introduce the dose of values as a reference dose and carry out corrective actions in order to decrease amount of dose in patients[10]. International Commission on Radiation Protection (ICRP) in 1990 recommended to optimize x-ray examinations for purpose of protecting patients against radiation according to optimized principle of reference dose or diagnostic reference level (DRL). In 1996, more details have been published on how to set conditions for using it. A DRL, as defined by International Commission on Radiological Protection (ICRP), is a form of practical attention level to an easily measured quantity and generally absorbed dose in air or tissue-equivalent material at the surface of a simple phantom or substitute a patient[11]. Application of diagnostic reference levels as an important optimization tool has been considered by many professional and corrector organizations including ICRP, American College of Radiology (ACR), American Association of Physicists in Medicine (AAPM), International Atomic Energy Agency (IAEA), the United Kingdom (U.K) and European Commission (EC). Applying DRL decreases overall patient dose in diagnostic radiology. For example, a dose reduction of 30% between 1984 and 1995 and an average dose reduction of 50% between 1985 and 2000 has been reported in the U.K.[12, 13]. It could be mentioned that Diagnostic Reference Level (DRL) attempts not only to decrease the dose of patient, but the aim of diagnostic reference levels (DRL) in radiology is to optimize radiation dose of patients while preserving diagnostic image quality and to find out abnormal high doses that do not confirm clinical results of a medical imaging examination significantly. It has been suggested that patient dose level or Diagnostic Reference Level (DRL) should be reviewed and further corrective measures should be taken. According to reports of International Atomic Energy Agency (IAEA), every country should have certain levels of diagnostic reference dose at national level. Then, it should define radiological protection program through comparing present patient dose with that of national diagnostic reference[14]. By 2001, the ICRP dose reference values, which were used to set relevant organizations in different countries, and advised to report diagnostic reference dose, should be adjusted for each country according to its actual conditions[15]. Today, these measurements are carried out every two years in some countries[16]. Iran is one of the countries that still have not provided clear and accurate information in this regard as essential considerations in this field. However, evaluations have been carried out in some provinces in Iran that the most brilliant studies have been conducted by Bahreyni Toosi MT et al and Keikhai Farzane et al[17, 18]. The present study has followed previous studies. Lack of databases in this regard indicates evaluation of patient dose and establishment of a local reference dose is one of the most important issues in this regard. Due to lack of national reference doses in Iran, obtained results from this study have been compared with previous studies conducted in Brazil (2004) and Slovenia (2005) and international reference levels[19, 20].

## Material And Methods

The present study was conducted in 13 public hospitals in order to evaluate radiation dose in twelve projections for conventional radiography in Mazandaran, Iran. Previously, annual quality control was performed for machines in these hospitals. At first, radiographic equipment information such as equipment manufacturer, model, year of installation, filtration, screen-film type and film speed were recorded. Afterwards, relevant data of patient and information on 12 projections in every center were collected. Film-Screen speed was about 400 in all study centers. Collection was analyzed and only films considered by radiographer were accepted for this study.

### Assessment of Patient Dose

Patient dose assessment was conducted in 12 projections including Skull (PA/AP), Skull (LAT), Cervical Spine (AP), Cervical Spine (LAT), Thoracic Spine (AP), Thoracic Spine (LAT) , Lumbar Spine (AP) , Lumbar Spine (LAT), Chest (PA/AP) , Chest (LAT) , Abdomen , and Pelvis. Required data for each projection were collected for 10 adult patients of both genders with average weight of 70±10 kg based on standard of National Board of Radiological Protection and then, related organizations were selected (IPSM 1992)[21]. In present study, ESAK (entrance surface air karma) and ESD (entrance skin dose) were selected for the purpose of determining patient doses and comparing them with international reference levels. It should be noted that ESD and ESAK are normal and common quantities for monitoring among adult patient’s dose in conventional radiography.

### Assessment of Determining Parameters for Adult Patients

Before determining patient dose, relevant information of determining parameters (kvp, mAs) and geometric parameters such as Focus to Skin Distance (FSD), Focus to Film Distances (FFD) and the size of film applied in this study were recorded. At least, 10 patients were included for each examination. An average weight for patients to 70±10 kg was practical as suggested[21, 22]. Obtained parameters in this section were later used to calculate patient doses in a tree-stage protocol including measurement of x-ray tube output, incident kerma measurements, and entrance surface air kerma values[23].

#### Measurement of X-ray Tube Output

In order to determine X-ray tube output, a dosimeter (Barracuda, RTI Electronic, Sweden), has been applied for measuring air kerma in energy ranges between 40-150 kvp. This type of semiconductor detector dosimeter, which was then connected to an electrometer, is a material with a low dispersion level (a flat cardboard plate). The distance from central axis beam focal spot x-rays was obtained 70cm. Radiation field size on the detector was 15×15cm[2] set up with a low scatter effect on the detector. In this case, tube voltage has been between 50 kvp and 110 kvp per every 10 steps. The desired mAs and radiation dose rates have been also recorded. This action was repeated three times for the same settings as mean dosimeter reading (in air) for kvp and mAs, respectively

### ESAK Calculation (Krema in the Air on Skin Surface)

As a result, ESAK was calculated through multiplying incident air kerma to patient’s skin surface in appropriate backscatter factor (BSF) coefficient. BSF was applied according to European Committee in relation to the tube potential and total  filtration devices, and radiation field size between 1.3 to 1.4 )ICRU 2005). In this study, the ratio rages between 1.28 and 1.30 variables applied for each calculation. Moreover, it should be noted that the amount of X-ray tube output should be modified at the input level of the patient’s body based on the distance of tube to detector while achieving tube output according to distance square reverse rule. The equation has been obtained as follows:

*ESAK*=* D_air_*× *BSF*× (*FDD*/*FSD*)^2^           (1)

*D_air_* is the value of dosimeter on (mGy), *BSF* is back scatter factor which is considered to 1.28 -1.30 in this investigation, *FDD* refers to distance of the focal spot to detector, and finally, *FSD* refers to the distance of x-ray focal spot to patient body[22]. Entrance Skin Dose (*ESD*) is a value to show energy given to skin and expression of tissue dose in place periphery Beam strikes with skin surface.

*ESD* value is obtained by producing *ESAK* in skin energy absorption coefficient to the air ratio, which is equal to 1.06. Equations 2 and 3 are presented as follows (this ratio is approximately 1.06 in conventional radiology in 110kvp, with ±1% error):

$$ESD\;ESAK=\;\left({\left(\frac{\mu_{en}}{\rho }\right)}_{water}/{\left(\frac{\mu_{en}}{\rho }\right)}_{air}\right)\;\;\;\;(2)$$

*ESD* = *ESAK* × 1.06                             (3)

## Results

The present study has been performed in 13 public hospitals for 15 radiography machines in Mazandaran, Iran for twelve common types of x-ray examinations. Table 1 shows the average skin dose resulting in twelve x-ray examination for 15 machines in 13 public hospitals. Parameters including minimum, mean, maximum, 3rd quartile, standard deviation, max/min ratio and ESAK have been presented in table 2 for each X-ray examination from distribution of ESD mean value for participating x-ray centers. Maximum/minimum ratio of ESD has demonstrated Variation of mean hospital dose for patients. Variation of mean patient dose in hospitals was obtained in high range. The range of mean hospital dose varies from 1.7 for AP lumbar spine to 10.2 for lateral skull.

**Table 1:** The mean skin dose the separated for different projections for each hospital.

**Room** **number**	**Skull (PA/AP)**	**Skull (LAT)**	**Cervical spine (AP)**	**Cervical spine (AP)**	**Chest (PA/AP)**	**Chest (LAT)**	**Thoracic spine (AP)**	**Thoracic spine (AP)**	**Lumbar spine (AP)**	**Lumbar spine (AP)**	**Abdomen**	**Pelvis**
**1**	1.61	1.42	0.475	0.673	0.82	1.75	2.25	2.77	2.37	3.6	0.983	1.05
**2**	0.711	0.619	0.470	0.50	0.266	0.953	1.41	1.62	1.60	3.86	1.10	0.766
**3**	0.453	0.305	0.255	0.342	0.388	1.06	1.70	1.91	1.95	3.78	0.670	0.648
**4**	0.518	0.246	0.363	0.375	0.353	0.833	1.59	2.27	2.62	3.42	1.44	1.43
**5**	1.93	1.40	1.26	1.28	0.704	1.49	3.10	4.14	3.86	5.88	3.97	3.02
**6**	1.49	0.815	0.742	0.797	0.418	0.855	2.68	3.20	2.70	3.90	1.52	2.05
**7**	1.97	1.32	1.12	1.17	0.437	0.917	2.20	3.10	2.27	4.70	3.00	2.75
**8**	3.06	2.57	1.07	1.08	1.45	1.89	3.27	3.90	3.55	4.45	4.00	3.65
**9**	2.05	1.18	1.05	1.46	0.648	0.797	3.12	3.46	3.21	4.08	2.41	2.4
**10**	0.633	0.598	0.250	0.340	0.255	0.915	2.48	3.53	3.30	5.87	3.78	3.00
**11**	3.00	2.55	1.00	1.05	0.416	0.864	2.62	2.98	3.20	4.18	2.41	2.39
**12**	1.09	0.698	0.50	0.953	0.33	0.830	2.20	3.73	3.00	4.20	3.00	2.70
**13**	1.68	1.30	0597	0.765	0.265	0.953	1.68	2.00	2.00	3.35	2.00	1.70
**14**	3.19	2.64	1.07	1.10	1.43	2.10	4.08	4.72	4.6	5.43	3.96	3.68
**15**	3.08	1.30	1.39	1.39	0.631	0.795	2.18	3.20	3.75	4.70	1.46	1.87

**Table 2:** ESAKa and ESDb(mGy) values  data for 12 examination types based on patient dose survey measurement

**Examination**	**Projection**	**Min**	**Mean**	**Max.**	**Standard** **deviation**	** 3^rd^** **Quartile**	**Max./Min. ratio**	** ESAK^a^**
Skull	PA or AP	0.45	1.47	3.19	0.98	3.05	7	1.38
Skull	LAT	0.24	1.01	2.57	0.79	1.42	10.2	0.99
Cervical Spine	AP	0.25	0.67	1.39	0.38	1.07	5.5	0.64
Cervical Spine	LAT	0.34	0.79	1.46	0.37	1.17	5.6	0.72
Chest	PA or AP	0.26	0.49	1.45	0.38	0.70	5.6	0.46
Chest	LAT	0.79	1.06	2.10	0.44	1.49	2.6	1.00
Thoracic Spine	AP	1.41	2.33	4.00	0.73	3.1	3	2.29
Thoracic Spine	LAT	1.62	3.00	4.72	0.82	3.73	3	2.84
Lumbar spine	AP	1.60	2.81	4.60	0.82	3.55	2.8	2.66
Lumbar spine	LAT	3.42	4.28	5.88	0.78	4.69	1.7	4.02
Abdomen	AP	0.67	2.07	3.97	1.17	3.78	6	1.95
Pelvis	AP	0.64	1.90	3.68	0.99	3	5.7	1.77

a: Entrance Surface Air Kerma

In table 3, range of tube potential, mAs, FFD and HVL used in all hospitals for each projection have been presented.  It should be mentioned that applied screen film has had speed of 400 in all hospitals.

**Table 3:** Range of exposure factors and Hvl used for each projection across all hospitals

**Examinations**	**Kvp**	**MAS**	**FFD**	**Hvl (mmAl)**
Skull PA/AP	67.2(60-78)	28.2(8-50)	90-110	2.1-3.5
Skull LAT	63.2(55-72)	25.4(6-50)	90-110	2-3.5
Cervical spine AP	61.86(55-68)	18.8(7-32)	90-110	2-3.2
Cervical spine LAT	64.13(56-68)	19.53(10-32)	90-115	2.1-3.3
Chest PA/AP	70(66-81)	15(6-32)	120-180	2-3.5
Chest LAT	79.9(72-90)	21.4(10-40)	120-180	2.4-3.5
Thoracic spine AP	69.7(59-75)	33.26(18-60)	90-110	2-3
Thoracic spine LAT	74(66-80)	39.8(30-60)	90-110	2-2.5
Lumbar spine AP	69.73(62-80)	39.2(20-60)	90-110	2.5-3.5
Lumbar spine LAT	82.1(72-95)	53.93(32-90)	90-110	2.5-3.5
Abdomen AP	71.1(72-95)	35.8(16-50)	90-110	2-3.2
Pelvis AP	68.8(66-72)	34.8(16-50)	90-110	2-3.5

**Figure 1:**
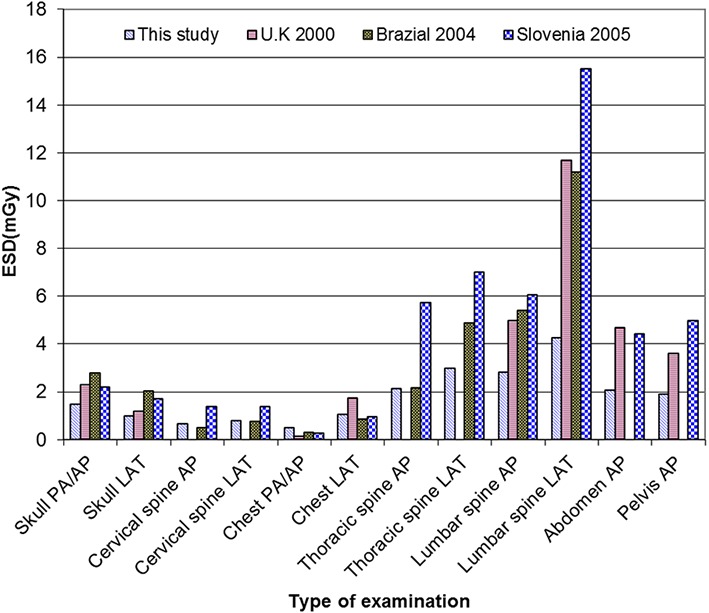
Comparison of the mean values of ESD (mGy) for the 12 projections in this study with reference levels from NRPB 2000(U.K) and Brazil and Slovenia

**Figure 2:**
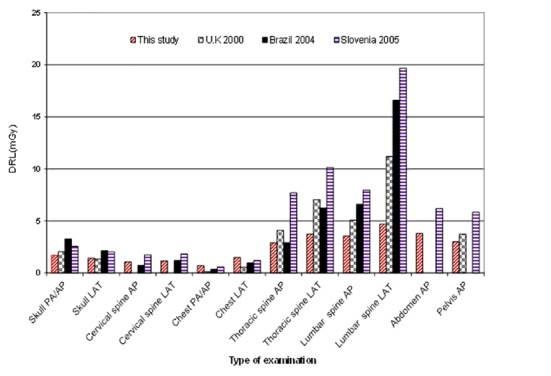
Comparison of the 3rdquartile (DRL) of ESD (mGy) for the 12 projections in this study with reference levels from NRPB 2000(U.K) and Brazil and Slovenia

In table 4, results of two similar studies conducted in Brazil and Slovenia [in columns 3 and 4] and also the average skin dose measured by NRBP 2000 [in column 2] have been presented for the purpose of comparison. figures 1 and 2 have illustrated comparison of mean values of ESD (mGy) and DRL for 12 projections in this study with reference levels from the U.K. (2000), Brazil (2004) and Slovenia (2005). Figure 1 indicates skin dose value for chest examinations in both projections is the same as values presented by the U.K. (2000) and also the amounts declared by Brazil and Slovenia. Figure 2 depicts comparison between values of diagnostic reference dose (DRL) obtained from this study and the values reported by the U.K. (2000), Brazil and Slovenia.

**Table 4:** The mean ESD and 3rdvalues of this study in compare with international value. Dash (*) indicates data not available

**Examination**	**This study**		** UK 2000[24] **		** Brazil 2004[19] **		** Slovenia 2005[20] **	
	Mean	3^rd^ quartile	Mean	3^rd^ quartile	Mean	3^rd^ quartile	Mean	3^rd^ quartile
Skull PA/AP	1.47	2.55	2.3	3	2.80	3.28	2.20	2.54
Skull LAT	1.01	1.42	1.2	1.5	2.04	2.14	1.73	2.02
Cervical spine(AP)	0.67	1.07	*	*	0.52	0.72	1.40	1.73
Cervical spine(LAT)	0.79	1.17	*	*	0.77	1.20	1.40	1.83
Chest PA	0.49	0.70	0.15	0.2	0.30	0.35	0.29	0.35
Chest LAT	1.06	1.49	0.75	1	0.87	0.96	0.96	1.20
Thoracic Spine(AP)	2.33	3.1	*	3.5	2.16	2.91	5.75	7.69
Thoracic Spine(LAT)	3	3.73	*	10	4.87	6.24	7.00	10.13
Lumbar Spine (AP)	2.81	3.55	5	6	5.4	6.6	6.06	7.98
Lumbar Spine (LAT)	4.28	4.69	11.7	14	11.2	16.6	15.52	19.67
Abdomen	2.07	3.78	4.7	4	*	*	4.43	6.18
Pelvis	1.90	3	3.6	6	*	*	4.99	5.83

## Discussion

The present survey has been conducted in 12 projection of routine x-ray examination in 13 public hospitals in Mazandaran province in Iran. The present study has been aimed at investigating the radiation dose for patients at conventional radiography examinations in Mazandaran and has also defined Diagnostic Reference Level (DRL) based on European Commission (EC) guidelines. All x-ray machines applied in this study were analogue and most of projections have been applied with anti-scatter radiation grid in these examinations. Obtained results from this study have shown wide variation in patient dose from 1.7 for an antero-posterior lumbar spine to 10.2 for the lateral skull, which have been presented in table 2. In order to determine Diagnostic Reference Level (DRL), the 3rd quartile of ESD derived from dose distribution for each examination is proposed quantity and the present study has applied the mentioned method[23]. Wide variation in patient’s dose among hospitals is demonstrated in table 1. The main reason for wide variations in dose is related to several factors such as patient weight, exposure factors, radiographic technique, FFD, film-screen speed, equipment type and processing performance. One possible reason for dose variation in the present study has been different; although in general it has been associated with high-dose hospital. The apparent difference in patient dose can be resulted from different conditions and methods of radiography applied in this survey. The initial comparison among patients’ dose in hospitals under study can depict that amount of patient dose in most of the examinations has been less than international reference levels. The exception has been related to chest radiography in both projections PA/AP and LAT, in which skin dose was more than obtained results from international references as the U.K. Probably, it could be because of higher mAs used in this study, compared to the U.K. The combination of film-screen used in this study was speed of 400; while the speed of film–screen of 200-400 was used in the study that has been done in the U.K. (2000). Application of film–screen speed of 200 can increase patient dose. Compared to Brazil and Slovenia, skin dose value in chest examination is greater than the amounts reported by mentioned countries. Although our recommended Diagnostic Reference Level (DRL) is lower than international references, wide range of dose distribution, especially in projection of lateral skull examination, indicated that routine radiography examination in Mazandaran should be optimized.

## Conclusion

The present study is determined to investigate wide variation in radiation dose of x-ray examinations among hospitals in Mazandaran, Iran. In order to reduce skin dose, an optimization procedure should be considered. Application of a reference dose (DRL) could be a practical method for this purpose. The role of optimization of radiography parameters for reducing patient dose is a significant issue. Through optimizing parameters, it would be possible to preserve image quality while reducing patient dose. For example, in chest radiography for both projections that dose values are higher than international levels, it is possible for skin dose to be reduced through increase in KVp and decrease in mAs. As a result, study of local diagnostic reference levels (LDRL) is so important that through this, users can compare their work and change their manner and choose convenient method in order to reduce dose levels for staff and patients. 

## References

[1] Benini A, International Atomic (1993). Medical radiation exposure. IAEA regional workshop radiation protection and quality assurance in diagnostic radiology.

[2] United Nations Scientific Committee on the Effects of Atomic Radiation (2000). Sources and effects of ionizing radiation: Report to the General Assembly, with scientific annexes. Annex D: Medical radiation exposures.

[3] Mettle FA, International Atomic (2001). Radiological risk associated with the various uses of radiation in medicine within the context of their associated benefits. Proceedings of international conferencee; 2001 March 26-30; Malaga, Spain.

[4] Rehani MM (1995). *Diagnostic imaging: Quality assurance*.

[5] Rehani MM, Kaul R, Kumar P, Berry M (1992). Quality assurance in diagnostic radiology. *Indian J Radiol*.

[6] Hart D, Hillier MC, Wall BF (2002). Doses to patients from medical x-ray examination in the UK: 2000 review; NRPB-W14.

[7] Gray JE, Archer BR, Butler PF, Hobbs BB, Mettler FA Jr, Pizzutiello RJ Jr (2005). Reference values for diagnostic radiology: application and impact. *Radiology*.

[8] International Commission on Radiological Protection (ICRP) (1982). Protection of the patient in diagnostic radiology. ICRP publication 34.

[9] Havukainen R, Pirinen M (1993). Patient dose and image quality in five standard x-ray examinations. *Med Phys*.

[10] Directorate-General for Research and Innovation, European Commission (1996). European guidelines on quality criteria for diagnostic radiographic images.

[11] Radiological protection (1996). A report of the International Commission on Radiological Protection. *Ann ICRP*.

[12] Hart D, Wall BF (2004). UK population dose from medical X-ray examinations. *Eur J Radiol*.

[13] Shrimpton PC, Wall BF, Hart D (1999). Diagnostic medical exposures in the UK. *Appl Radiat Isot*.

[14] Muhogora WE, Ahmed NA, Almosabihi A, Alsuwaidi JS, Beganovic A, Ciraj-Bjelac O (2008). Patient doses in radiographic examinations in 12 countries in Asia, Africa, and Eastern Europe: initial results from IAEA projects. *AJR Am J Roentgenol*.

[15] Directorate-General for Research and Innovation, European Commission (1999). Radiation Protection 109.Guidance on Diagnostic reference levels (DRL) for Medical Exposures.

[16] Berni D, Gori C, Lazzari B, Mazzocchi S, Rossi F, Zatelli G (2002). Use of TLD in evaluating diagnostic reference levels for some radiological examinations. *Radiat Prot Dosimetry*.

[17] Toosi MT, Asadinezhad M (2007). Local diagnostic reference levels for some common diagnostic X-ray examinations in Tehran county of Iran. *Radiat Prot Dosimetry*.

[18] Farzaneh MJK, Shandiz MS, Vardian M, Deevband MR, Kardan MR (2011). Evaluation of Image Quality and Patient Dose in Conventional Radiography Examinations in Radiology Centers in Sistan and Baluchestan, Iran and Comparing with that of International Guidelines Levels2011. *Indian J Sci Technol*.

[19] Freitas MB, Yoshimura EM (2009). Diagnostic reference levels for the most frequent radiological examinations carried out in Brazil. *Rev Panam Salud Publica*.

[20] Skrk D, Zontar D, Zdesar U (2006). Diagnostic reference levels for X-ray examinations in Slovenia. *Radiol Oncol*.

[21] Institute of Physical Sciences in Medicine (IPSM), National Radiological Protection Board (NRBP), Collage of Radiographers (1992). National protocol for patient dose measurements in diagnostic radiology.

[22] (2005). Patient dosimetry for x rays used in medical imaging. *J ICRU*.

[23] Hart D, Shrimpton PC (1991). The significance of patient weight when comparing X-ray room performance against guideline levels of dose. *Br J Radiol*.

[24] Hart D, Hiller MC, Wall B (2002). Doses to patients from medical X-ray examinations in the UK – 2000 Reviw. Report NRBP.

